# Supplementary material for: Causal Relationship between Aging and Anorexia Nervosa: A White-Matter-Microstructure-Mediated Mendelian Randomization Analysis
Source: Biomedicines. 2024 Aug 16;12(8):1874. doi: 10.3390/biomedicines12081874 (PMC11351342; doi:10.3390/biomedicines12081874)
Supplement: Supplementary file 1 [file biomedicines-12-01874-s001.zip › Supplementary Figure/Figure S4/Singe SNP plot of MR analysis for TL to ubm-b-1855.pdf]

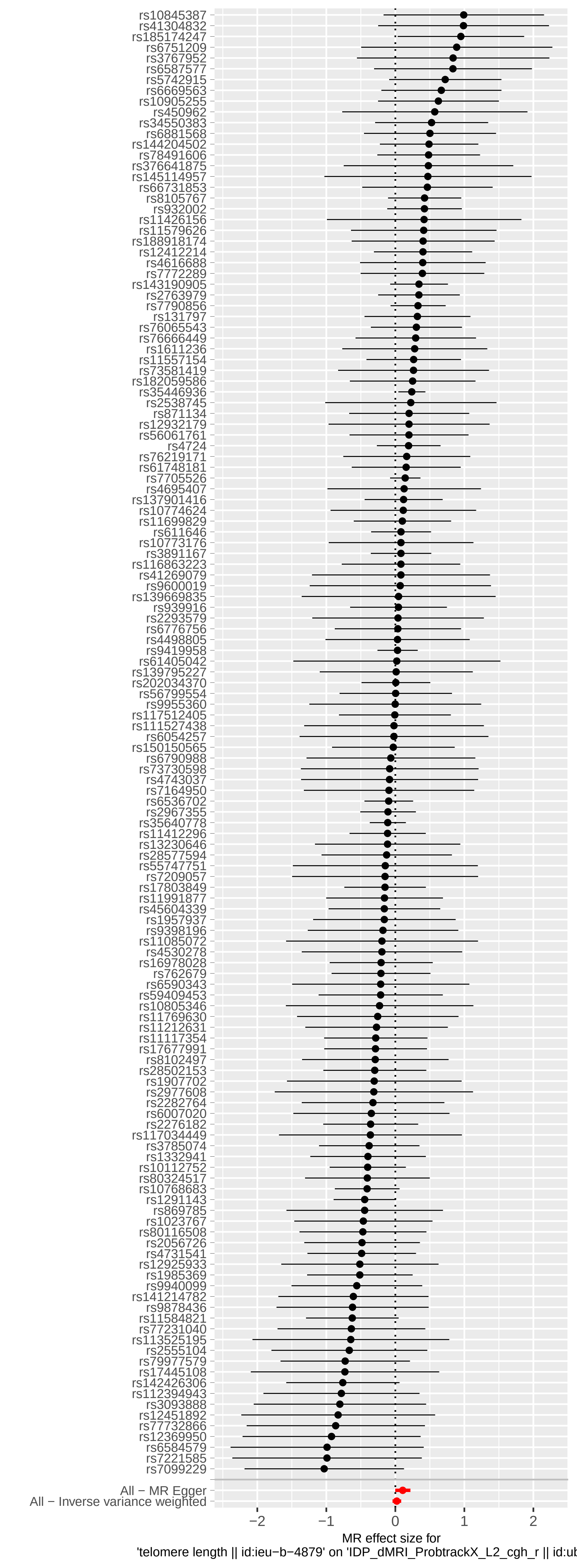
rs10845387
rs41304832
rs185174247
rs6751209
rs3767952
rs6587577
rs5742915
rs6669563
rs10905255
rs450962
rs34550383
rs6881568
rs144204502
rs78491606
rs376641875
rs145114957
rs66731853
rs8105767
rs932002
rs11426156
rs11579626
rs188918174
rs12412214
rs4616688
rs7772289
rs143190905
rs2763979
rs7790856
rs131797
rs76065543
rs76666449
rs1611236
rs11557154
rs73581419
rs182059586
rs35446936
rs2538745
rs871134
rs12932179
rs56061761
rs4724
rs76219171
rs61748181
rs7705526
rs4695407
rs137901416
rs10774624
rs11699829
rs611646
rs10773176
rs3891167
rs116863223
rs41269079
rs9600019
rs139669835
rs939916
rs2293579
rs6776756
rs4498805
rs9419958
rs61405042
rs139795227
rs202034370
rs56799554
rs9955360
rs117512405
rs111527438
rs6054257
rs150150565
rs6790988
rs73730598
rs4743037
rs7164950
rs6536702
rs2967355
rs35640778
rs11412296
rs13230646
rs28577594
rs55747751
rs7209057
rs17803849
rs11991877
rs45604339
rs1957937
rs9398196
rs11085072
rs4530278
rs16978028
rs762679
rs6590343
rs59409453
rs10805346
rs11769630
rs11212631
rs11117354
rs17677991
rs8102497
rs28502153
rs1907702
rs2977608
rs2282764
rs6007020
rs2276182
rs117034449
rs3785074
rs1332941
rs10112752
rs80324517
rs10768683
rs1291143
rs869785
rs1023767
rs80116508
rs2056726
rs4731541
rs12925933
rs1985369
rs9940099
rs141214782
rs9878436
rs11584821
rs77231040
rs113525195
rs2555104
rs79977579
rs17445108
rs142426306
rs112394943
rs3093888
rs12451892
rs77732866
rs12369950
rs6584579
rs7221585
rs7099229
All − MR Egger
All − Inverse variance weighted
−2
−1
0
1
2
MR effect size for
'telomere length || id:ieu−b−4879' on 'IDP_dMRI_ProbtrackX_L2_cgh_r || id:ub
